# Knowledge, attitudes and practice pertaining to depression among primary health care workers in Tanzania

**DOI:** 10.1186/1752-4458-3-5

**Published:** 2009-02-25

**Authors:** Joseph Mbatia, Ajit Shah, Rachel Jenkins

**Affiliations:** 1 Ministry of Health, Dar es Salaam, Tanzania; 2 Institute for Philosophy, Diversity and Mental Health, Centre for Ethnicity and Health, University of Central Lancashire, Preston, UK; 3 WHO Collaborating Centre for Research and Training in Mental Health and Section of Mental Health Policy, Health Service and Population Research Department, PO35, David Goldberg Centre, Institute of Psychiatry, De Crespigny Park, London, SE5 8AF, UK

## Abstract

**Background:**

Examination of consultation data in a variety of primary care settings in Tanzania shows that, while psychoses are routinely diagnosed and treated at primary care level, depression is rarely recorded as a reason for consultation. Since, epidemiological studies elswhere show that depression is a much more common disorder than psychosis, a series of studies were undertaken to elucidate this apparent paradox in Tanzania and inform mental health policy; firstly, a household prevalence study to ascertain the prevalence of common mental disorders at community level in Tanzania; secondly, a study to ascertain the prevalence of common mental disorders in primary care attenders; and thirdly, a study to ascertain the current status of the knowledge, attitude and practice pertaining to depression among primary health care workers. This paper reports the findings of the latter study.

**Methods:**

All the primary health care workers (N = 14) in four primary health care centres in Tanzania were asked to complete the Depression Attitude Questionnaire, which assesses the health worker's knowledge and attitude towards the causes, consequences and treatment of depression.

**Results:**

The majority of respondents felt that rates of depression had increased in recent years, believed that life events were important in the aetiology of depression, and generally held positive views about pharmacological and psychological treatments of depression, prognosis and their own involvement in the treatment of depressed patients.

However, the majority of respondents felt that becoming depressed is a way that people with poor stamina deal with life difficulties.

**Conclusion:**

The findings suggest a need to strengthen the training of primary health care workers in Tanzania about the detection of depression, pharmacological and psychological treatments, and psychosocial interventions.

## Background

Human resources devoted to health systems in sub-Saharan Africa are scarce. In order to bring health care to the population, strategic primary care structures have evolved, although there is widespread concern about their performance and capacity to deliver in resource poor settings. Primary care of mental disorders is crucial in all parts of the world because of the sheer scale of psychiatric morbidity, and specially in sub-Saharan Africa where specialist expertise is very scarce. Tanzania only has 13 psychiatrists for a population of 37 million, and nine of these psychiatrists are in Dar es Salaam. In fact Tanzania has produced a total of 25 psychiatrists, of whom 11 are working in the Tanzanian public service, 2 work in private practice, 9 are aborad and 2 have retired. In addition, 1000 mental health nurses have been trained, but only 460 are currently deployed in the mental health services.

Examination of consultation data in primary care settings in Tanzania shows that while psychoses are routinely diagnosed and treated at primary care level, depression is rarely recorded as a reason for consultation. Since depression is much more common than psychosis, a series of studies were intiated to elucidate the situation and inform mental health policy in Tanzania. Firstly a household prevalence study was initiated to ascertain the prevalence of mental disorders at community level, secondly a study to ascertain the prevalence of common mental disorders in primary care attenders and thirdly a study to ascertain the current status of the knowledge, attitude and practice pertaining to depression among primary health care workers. This paper reports the findings of the latter study.

The Tanzanian primary health care system has several levels or tiers. The first tier consists of volunteer village health workers, who each look after about 50 houses, have a first aid box, and are taught to give injections, wash and dress wounds etc. The second tier is the dispensary level, each covering about 10,000 population and staffed by rural medical aides and clinical officers, who are primary school leavers with a period of 3 years training in assessment, diagnosis and management of some general physical health conditions (e.g. malaria, maternal health, tuberculosis, leprosy, parasitic infections and other infectious diseases). Thus the catchment area of each dispensary might be expected to have at least 50 people with schizophrenia, 50 people with bipolar illness, 250–400 people with severe depression, and 50 people with epilepsy. However, in practice a large proportion of people consult traditional healers first [1-3], the rural medical aides lack the necessary medical skills to assess and treat, and do not have appropriate psychotropic medications. Dispensaries may also have one bed for overnight observations. The third tier is the health centre level, covering a population of 120,000 to 150,000, of which there are 3 or 4 in a district. They have a clinical officer and qualified nurses who may also have additional training in midwifery, psychiatry, opthalmology, ENT etc. These health centres essentially function as out-patient clinics and may also have 2–4 inpatients beds, where patients may be treated for 2 to 3 days before discharge or referral to a district hospital. However, these facilities are not evenly distributed and in some areas this service is provided by non-governmental organisations rather than the state. The fourth tier is the district hospital.

There have been very few prevalence studies of mental disorders in primary care settings in Tanzania.

A Tanzanian study of primary health care attenders (tier 3) and those consulting traditional healers, using the Clinical Interview Schedule (Revised) [4], reported prevalence rates of common mental disorders as 24% and 48% in these two settings respectively [5]. The prevalence rates of depression, mixed anxiety-depressive disorder, phobias and obsessive compulsive disorders among primary care attenders were 3.3%, 15.2%, 7.3% and 5.1% respectively; the corresponding figures in those consulting traditional healers were 14.2%, 27.8%, 2.2% and 5.1% [5]. A population-based study in Zanzibar, one of the two political units of the United Republic of Tanzania, reported a prevalence rate of 0.55% of mental disorders, but no cases of depression were identified [1]. However, the low prevalence of mental disorders and the absence of depression was likely to have been an artefact of the method of case ascertainment, whereby household members were asked to identify those with mental disorders on the basis of a description of symptoms, and this approach was heavily biased towards presence of behaviour disturbance and psychosis. Twenty percent of referrals to a specialist mental health unit in Dar es Salaam had neurotic (non-psychotic common mental) disorders [6]. There have been no previous studies of attitudes to depression amongst primary care attenders in Tanzania.

The National Mental Health Pilot Programme in Tanzania, [7,8], aimed to provide seamless mental health care at all tiers of primary care through to specialist psychiatric services and raise the awareness of mental health in communities. It informed the Tanzanian government's overall health policy of 1990 which included mental health in primary health care and made provision for the basic primary care health structure to diagnose and treat mental disorders.

However, due to lack of government financing in subsequent years, it was not possible to sustain the implementation of this policy across Tanzania over the ensuing years, until in 2002/3 when mental health has now been included in the national health sector strategic plan, enabling some continuing professional development for primary care to restart. This paper therefore reports a study of four pilot health clinics in urban Dar es Salaam, which had not been part of the earlier National Mental Health Pilot (held in Kilimanjaro and Morogoro regions). The study was conducted prior to the re-initiation of the continuing professional development programme for primary care.

## Methods

### Study population

The study population in Tanzania consisted of all health workers employed in four primary health care clinics (third tier), selected as a convenience sample, in urban Dar es Salaam. The health workers were ten nurses with a three year or four year general nurse training, and four assistant medical officers with a three year training. They had received a short mental health module in their basic training, received according to the Tanzanian national curricula, but had not at that time received any mental health continuing professional development (CPD) since their basic training. This situation was similar to that pertaining across East Africa and indeed sub Saharan Africa, although there are now systematic efforts to provide mental health CPD across several regions of Tanzania, and some other countries including a national programme in Kenya, and a pilot programme in Malawi (all to be reported elsewhere). Staff numbers were low in the four clinics since health workers are scarce in sub Saharan Africa. We studied staff attitudes in these four clinics as part of a wider study (to be reported elsewhere) which also examined the GHQ prevalence of attenders in those clinics over a representative week.

### Study instrument

The Depression Attitude Questionnaire (DAQ) [9], is a self completion questionnaire which assesses health workers knowledge and attitude towards the causes, consequences and treatment of depression. Each question has four possible responses: strongly disagree, moderately disagree, moderately agree and strongly agree. The DAQ was translated into Swahili by a psychiatrist and back translated into English by a sociologist to check for accuracy for use in the Tanzanian context. The back translation confirmed the accuracy of the original translation into Swahili. Some questions were adapted for local circumstances. Although it was originally devised for use in the United Kingdom, it has been successfully used in a number of other countries, including Brazil [10], and Abu Dhabi [11].

### Study Procedure

The primary health care workers were asked to complete the DAQ on a specified day.

### Data analysis

The data was transcribed on to an SPSS sheet, entered into a computerised database and analysed using SPSS. Simple descriptive statistics were used to describe the responses to the questions on the DAQ. For the purpose of analysis the categories of strongly disagree and moderately disagree were collapsed into a single category of disagree; the same was repeated for the two categories of agree.

## Results

All 14 primary health care workers in the four primary care clinics in Tanzania completed the DAQ. All the primary health care workers were between the ages of 40 years and 55 years (except one whose age was 27 years), half were males and half were females. Table 1 shows the responses to the DAQ questions.

**Table 1:** Tanzanian responses to DAQ

**DAQ question**	**Disagree N (%)**	**Agree N (%)**	**Dont' know N (%)**
During the last 5 years I have seen an increase in the number of patients presenting with depressive symptoms	1 (7.1)	13 (92.9)	0 (0)

The majority of depression we see originated from recent misfortune	3 (21.4)	10 (71.4)	1 (7.1)

Most depressive disorders improve without medication	6 (42.8)	7 (50)	1 (7.1)

Biochemical abnormality is at the basis of more severe depression	5 (35.7)	9 (64.3)	0 (0)

Difficult to differentiate unhappiness or a clinical depressive disorder that needs treatment	9 (64.3)	4 (28.6)	1 (7.1)

It is possible to distinguish two groups of depression, one psychological in origin and the other caused by biochemical mechanisms	3 (21.4)	11 (78.6)	0 (0)

Becoming depressed is a way that people with poor stamina deal with life difficulties	5 (35.7)	9 (64.3)	0 (0)

Depressed patients are more likely to have experienced deprivation in early life than other people	8 (57.1)	4 (28.6)	2 (14.3)

I feel comfortable dealing with depressed patients	1 (7.1)	13 (92.9)	0 (0)

Depression reflects a characteristic response which is not amenable to change	9 (64.3)	3 (21.4)	2 (14.3)

Becoming depressed is a natural part of becoming old	9 (64.3)	5 (35.7)	0 (0)

The primary health care worker could be a useful person to support depressed patients	3 (21.4)	11 (78.6)	0 (0)

Working with depressed patients is heavy going	5 (35.7)	8 (57.1)	1 (7.1)

There is little to be offered to depressed patients who do not respond to what primary health care workers do	11 (78.6)	3 (21.4)	0 (0)

It is rewarding to look after depressed patients	4 (28.6)	9 (64.3)	1 (7.1)

If depressed patients need antidepressants, they are better off with psychiatrists than with primary health care workers	8 (57.1)	6 (42.9)	0 (0)

Antidepressants usually produce a satisfactory result in the treatment of depressed patients in general practice	2 (14.3)	11 (78.6)	1 (7.1)

Psychotherapy for depressed patients should be left to a specialist	4 (28.6)	10 (71.4)	0 (0)

If psychotherapy were freely available, this would be more beneficial than antidepressants for most depressed patients	2 (14.3)	12 (85.7)	0 (0)

### Rates of depression

The vast majority of primary care workers perceived an increase in the rates of depression in the last five years.

### Stigmatising attitudes to depression

Almost two-thirds of the sample believed that becoming depressed is a way that people with poor stamina deal with life difficulties, but almost two thirds disagreed with the statement that becoming depressed is part of growing old.

### Ability to diagnose depression and categorising depression

Two thirds did not feel they had difficulties in differentiating between unhappiness and a clinical depressive disorder that requires treatment, and, the majority of primary care workers felt that it is possible to distinguish two main groups of depression, one psychological in origin and the other caused by biological mechanisms.

### Causes of depression

The vast majority of primary care workers acknowledged the role of life events in the development of depression.; however, only 29% believed that depressed patients are more likely to have experienced deprivation early in life. The majority of primary care workers believed that biochemical abnormality was the basis of severe depression.

### Attitudes to treating depression

The vast majority of primary care workers felt comfortable dealing with depressed patients and felt that they could be a useful person to support depressed patients. The majority of primary care workers felt that it was rewarding to work with depressed patients, but found working with depressed patients "heavy going". Two-thirds of primary care workers disagreed with the statement that there is little to be offered to those depressed patients who do not respond to treatment from them.

### Treatment of depression

Half of the sample disagreed with the statement that depressive disorders improve without medication. The vast majority of primary care workers believed that psychotherapy would be more beneficial than antidepressants for most depressed patients. Only 40% of primary health care workers believed that if depressed patients need antidepressants, they would be better off with psychiatrists than community health workers; but 70% primary care workers agreed with the statement that psychotherapy for depressed patients should be left to specialists.

### Prognosis of depression

Over two-thirds of primary care workers disagreed with the statement that depression reflects a characteristic response which is not amenable to change.

Almost four-fifths of primary care workers felt that antidepressants usually produce a satisfactory result in the treatment of depressed patients in general practice.

## Discussion

This is the first study of attitudes, knowledge and practice about depression in primary care workers in Tanzania, and it found that the majority of respondents felt that rates of depression had increased in recent years, believed that life events were important in the aetiology of depression, and generally held positive views about pharmacological and psychological treatments of depression, prognosis and their own involvement in the treatment of depressed patients.

The study is limited by the small size of the sample of primary care workers, and by the fact that they were all working in Dar es Salaam, so the results may not be generalised to the rest of the country.

It is not known whether rates of depression are in fact increasing, but we have conducted a baseline study of common mental disorders in urban Tanzania (Jenkins et al 2009-submitted to JAMA) so it will be possible to repeat this and assess prevalence trends. Indeed, it is possible that rates are not increasing susbstantially, but rather that awareness of depression is rising in the general population and in health workers.

The majority of respondents reported felt that becoming depressed is a way that people with poor stamina deal with life difficulties. The finding that the majority of the sample felt that it is possible to distinguish two main groups of depression, one psychological in origin and the other caused by biological mechanisms, and only a third reported difficulties in differentiating between unhappiness and a clinical depressive disorder that requires treatment, is in fact somewhat better than observations among general practitioners in other countries [10].

The majority of the sample did however hold some stigmatising attititudes about depression, (believing that becoming depressed is a way that people with poor stamina deal with life difficulties) which may contribute to the difficulty which some experience in differentiation between unhappiness and clinically significant depressive illness. However, it was encouraging that the vast majority of primary care workers disagreed that becoming depressed is part of growing old. It was also encouraging that the vast majority of primary care workers acknowledged the role of life events in the development of depression, and nearly a third believed that depressed patients are more likely to have experienced deprivation early in life than other people; The aetiological importance attached to life events and deprivation early in life is consistent with previous studies in East Africa [12-15], and training in the detection and treatment of mental illness should also incorporate the increasing body of literature on risk factors and life events related to the development of depression in Kenya and Tanzania. Food insecurity was associated with depression and anxiety in four ethnic groups in rural communities in Tanzania [16]. The majority belief that biochemical abnormality is the basis of severe depression is consistent with similar observations among general practitioners in other countries [5].

The attitudes of the sample to treatment were mixed, It was encouraging that over two-thirds of the primary care workers on Tanzania mainland disagreed with the statement that depression is not amenable to change, and this is consistent with observations in Zanzibar in a mixed sample, including health workers and traditional healers [2].

About half of the primary care workers disagreed with the statement that depressive disorders improve without medication, and the vast majority felt that antidepressants usually produce a satisfactory result in the treatment of depressed patients in primary care, a finding consistent with observations among general practitioners in other countries [5,10]. Nevertheless, three-fifths of patients referred to a Tanzanian specialist psychiatric clinic had previously received inadequate treatment with psychotropics and 17% of patients had received treatment with antimalarials for their mental illness [5]. Fourty percent of primary health care workers belived that if depressed people need antidepressants they would be better off with psychiatrists than community health workers, and this has been observed among general practitioners in other countries [10].

As found in general practitioners in other countries [5], the vast majority of primary care workers believed that psychotherapy would be more beneficial than antidepressants for most depressed patients. Nonetheless four-fifths of primary care workers agreed with the statement that psychotherapy for depressed patients should be left to specialists, and this is also consistent with observations among general practitioners in other countries [10].

It is encouraging that the majority of primary care workers in Tanzania felt comfortable dealing with depressed patients, that they could be a useful person to support depressed patients, that it was rewarding to work with depressed patients, and they disagreed that there is little to be offered to those depressed patients who do not respond to treatment from them.

However, the majority of primary care workers in Tanzania found working with depressed patients "heavy going". These findings have been observed among general practitioners in other countries [5,10]. This paradox of high prevalence rates, toegther with low levels of treatment but nonetheless generally positive attitudes has been found in a number of countries [17], and can be addressed by a systematic approach as set out in the recent WHO Guidance on intergation of mental health in primary care [18].

## Conclusion

Depressive illness is under-diagnosed and under-treated in primary health care setting throughout the world [17]. The generally positive attitudes towards depression found in this study of primary care workers in Tanzania may be capitalised on in the training and continuing professional development of primary health care workers in the detection and management of depression. This is now being conducted across several regions of Tanzania, and across all regions of Kenya, both through structured one week ministry of health programmes, accompanied by provision of WHO primary care guidelines, tailored for each country, medicine supply to the clinics, incusion of mental health in health management information systems, and regular supervision from the district level, funded by the national health sector reforms.

## Competing interests

The authors declare that they have no competing interests.

## Authors' contributions

The project was conceived and designed by RJ who wrote the first draft of the paper, data was collected by JM, analysed by AS and all authors contributed to the final draft.
